# Strengthening pre-service training of healthcare workers on immunisation and effective vaccine management: the experience of Kenya Medical Training College

**DOI:** 10.11604/pamj.2022.41.47.30502

**Published:** 2022-01-18

**Authors:** Margaret Juma, Balcha Masresha, Adebayo Adekola, Carine Dochez

**Affiliations:** 1 Kenya Medical Training College, Nairobi, Kenya,; 2 World Health Organization, Regional Office for Africa, Brazzaville, Congo,; 3 UNICEF Supply Division, Copenhagen, Denmark,; 4 Network for Education and Support in Immunisation, University of Antwerp, Antwerp, Belgium

**Keywords:** Kenya, immunisation programme, education, curriculum, vaccines, communication

## Abstract

Pre-service health training institutions have a key role in training qualified medical and nursing staff deployable in immunisation programmes, making them capable of addressing complex situations, sustaining routine immunisation and introducing new vaccines and technologies. The incorporation of immunisation-related content into nursing and midwifery education is essential to improve and strengthen immunisation service delivery, disease surveillance, logistics, communication and management practices. Clinical and public health training incorporating learning objectives on immunisation that are specific to the Expanded Programme on Immunisation (EPI), will enable students to develop a firm basis of core knowledge and skills in immunisation. To assist health training institutions in the African Region and to facilitate the systematic revision of EPI curricula, two prototype curricula, one for medical and one for nursing/midwifery schools, were developed by WHO/AFRO, NESI/University of Antwerp and other partners in 2006 and revised in 2015. Kenya Medical Training College (KMTC) has been at the forefront in revising and updating their institutional EPI curriculum for the pre-service Kenyan Registered Community Health Nursing programme based on the EPI prototype curriculum. Building on the successful strengthening of the EPI curriculum, KMTC will now embark on improving education and training for effective vaccine and cold chain management for selected training programmes. The different steps taken by KMTC to strengthen EPI teaching and learning can support other health training institutions who are willing to integrate the content of the EPI prototype curriculum in their own institutional curricula by adapting them to the local context.

## Introduction

Pre-service health training institutions are key in training competent medical and nursing staff deployable in immunisation programmes capable of addressing complex situations, sustaining routine immunisation and introducing new vaccines and technologies. Nurse lecturers are tasked to educate and train students on relevant knowledge on the Expanded Programme on Immunisation (EPI), skills and attitudes towards immunisation. Such training should be competency based and integrated throughout the curriculum [1]. Student achievement is highly influenced by the quality of teaching; therefore, well-educated nurse lecturers are of paramount importance [2].

Training needs assessments conducted at health training institutions in 12 target countries in the African Region between 2002-2004, identified gaps in pre-service training, including: incomplete or outdated EPI content in the pre-service curricula; lack of demonstration equipment and up-to-date EPI reference material; insufficient time allocation for EPI theory; as well as insufficient knowledge on current EPI theory and practice of lecturers [3]. Similar results were found in a study conducted in Nigeria that assessed the EPI knowledge and skills of tutors in 5 pre-service health training institutions [4].

The incorporation of EPI content into undergraduate training programmes is important for strengthening immunisation service delivery, logistics, surveillance, communication and management practices. Healthcare workers have a central role in maintaining public trust in vaccination through direct communication with the vaccinated person or the caregiver of the child. Therefore, healthcare workers need to be knowledgeable about vaccines and immunisation programmes and have good communication skills. In this way, healthcare workers can contribute to improve public confidence in the immunisation services provided, creating sustained demand leading to high immunisation rates.

The knowledge and skills of the healthcare worker can be reinforced through refresher training. Evidence exists that in-service interactive workshops and courses improve the performance of healthcare workers [5-7], while pre-service clinical and public health training incorporating the learning objectives of EPI will enable students to develop a firm basis of EPI core knowledge and skills in line with new developments and strategies in the fast-moving area of vaccines and immunisation. Linking professional education with service reality is key to the overall success.

## Methods

To assist health training institutions in the African Region with the systematic revision of EPI curricula, two prototype curricula, one for medical and one for nursing/midwifery schools, were developed by WHO/AFRO, NESI/University of Antwerp and other partners in 2006 and revised in 2015, and are available in English and French [8, 9]. The general objective of the prototype curricula is to strengthen the teaching and learning processes of immunisation within the existing curriculum for pre-service education programmes for medical doctors and nurses/midwives.

The first part of the prototype curriculum describes the exit profile and core competencies required for immunisation service providers, including the EPI manager, disease surveillance officer/epidemiologist, cold chain officer, logistics officer, communication/social mobilization/health promotion officer and data manager/statistician. The expected competencies and skills in immunisation for a newly qualified nurse/midwife and a district EPI nurse are outlined in detail. The second part of the curriculum describes the proposed content topics of the EPI curriculum (Table 1). Each topic is divided into sub-topics, indicating the learning objectives, time required to cover the topic, type of practical exercises and how to organize them, and recommended reference materials.

**Table 1:** content topics of the EPI prototype curriculum for nursing/midwifery schools (WHO, 2015)

Content topics		Theory		Practical session		Field placement	
		Nurse/midwife	Registered nurse/midwife	Nurse/midwife	Registered nurse/midwife	Nurse/midwife	Registered nurse/midwife
1	Immunization systems and operations	45	45				
2	Immunization policies, norms and standards	30	30				
3	Immunization service delivery strategies and innovative approaches	60	60				
4	Target diseases for immunization programmes and disease surveillance	60	60	3	3	1 week	2.5 days
5	Vaccinology and the Expanded Programme on Immunization vaccines	45	60				
6.1	General guidelines for vaccine administration	45	45				
6.2	How to administer EPI vaccines and vitamin A	60	60	3	3		
6.3	Cold chain and vaccine handling - logistics support	60	60	6	6	2 days	1 week
6.4	Immunization safety	60	60				
6.5	How to organize an immunization session	60	60				
6.6	Conducting an immunization session	60	60	6	6	2 weeks	2 weeks
6.7	Communication for immunization programmes	60	60			1 day	2.5 days
7.1	Introduction to immunization programme management	30	45				
7.2	Planning immunization activities and financial management	60	90				
7.3	Supervision by programme managers	30	45				
7.4	Monitoring of immunization programmes and data management	60	60		3	1 day	2.5 days
7.5	Evaluation of immunization programmes	45	60				
TOTAL TIME ALLOCATION TO EPI (theory and field placement)		870 hrs	960 hrs	18 hrs	21 hrs	3 weeks 4 days	4.5 weeks

https://www.nesi.be/wp-content/uploads/2020/05/ImmunizationCurNursing-eng-1.pdf

The EPI prototype curricula have been widely distributed to health training institutions within countries of the African Region. In 2011, WHO/AFRO and NESI/University of Antwerp carried out an evaluation in nine African countries to assess the status of introduction of the EPI prototype curricula in health training institutions. A total of 61 health training institutions were visited, including 16 medical schools and 45 nursing/midwifery schools (unpublished information, available from NESI and WHO/AFRO). Kenya Medical Training College (KMTC) was one of the six health training institutions in Kenya that participated in the evaluation. With its network of 71 campuses, strategically located across the country in 45 out of 47 counties, KMTC is one of the leading health training institutions in Kenya, and the driving force advocating for updating EPI curricula at all pre-service nursing/midwifery training institutions in the country. All private and public nurse training institutions in Kenya follow a standard EPI syllabus which has been updated and approved by the Nursing Council of Kenya (NCK) in 2011, incorporating EPI priority areas.

## Results

**Figure 1:**
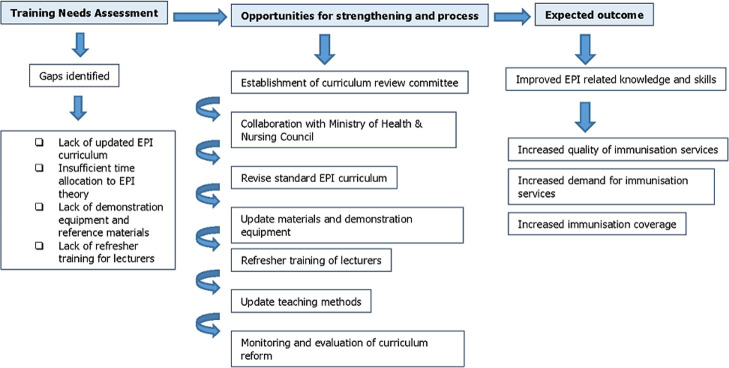
process of EPI curriculum revision and expected outcome

Since 2013, the KMTC EPI focal person has been part of the WHO/AFRO working group to revise the EPI prototype curriculum for nursing/midwifery schools, which was finalised in 2015. KMTC was committed to immediately implement the revised curriculum with the support from NESI/University of Antwerp. KMTC management together with the College Curriculum Review Committee supported the EPI focal person to review the institutional pre-service curriculum to incorporate the new content of the EPI prototype curriculum (Figure 1).

Following a survey to determine the technical competences (knowledge, skills and attitudes) of nurse lecturers in EPI at the different KMTC colleges, a workshop was organised to strengthen the teaching and learning of immunisation within the existing curriculum for the pre-service Kenyan Registered Community Health Nursing (KRCHN) programme. Consensus was obtained on the EPI content to be incorporated in the KRCHN curriculum as guided by NCK and in line with the EPI prototype curriculum. Following consensus on the EPI content, an EPI learning guide to be used by students during the skills laboratory sessions has been developed. In the revised curriculum, the time allocation has been increased from 12 hours to 24 hours EPI theory. Practical training includes 6 hours of demonstrations in a skills laboratory, and an integrated clinical placement of 28 weeks in a Maternal, Child Health and Family Planning Clinic.

During the final step, refresher training is organised for all KMTC nurse lecturers, using the recently revised Mid-Level Management (MLM) modules as the standard training materials [6], in order for them to deliver the updated EPI content with confidence.

## Discussion

A summary of the different key steps taken for strengthening EPI pre-service training at KMTC is listed here: KMTC participation in the WHO/AFRO and NESI pre-service training evaluation; receipt of evaluation report by WHO/AFRO and NESI; receipt of WHO EPI prototype curriculum; sensitization of KMTC managers on EPI prototype curriculum; sensitization of KMTC curriculum review committee on EPI prototype curriculum; sensitization of NCK on EPI prototype curriculum; adoption of the EPI prototype curriculum content in the Syllabus of the NCK Kenya Registered Community Health Nursing; adoption of the EPI prototype curriculum by the Department of Nursing to be used for teaching content while awaiting due curriculum review process; refresher training organised for pre-service nurse lecturers at KMTC and private institutions, including induction on the EPI prototype curriculum, using MLM training modules; review of KMTC Pre-service Nursing Curriculum. Currently pre-service Immunisation Module includes 24 hours theory, 6 hours Skills Laboratory practical demonstrations and 28 weeks of Integrated Clinical Placement

The introduction of the revised EPI curriculum presents a good opportunity to strengthen capacity for Effective Vaccine Management (EVM) which is required for routine immunisation and deployment of new vaccines, including COVID-19 vaccine. This will ultimately contribute to maintaining adequate vaccine stock levels, reducing wastage, preventing cold chain breakdown and forecasting for immunisation supplies across Kenyan counties.

Building on the experience and successful revision of the EPI curriculum, KMTC in collaboration with partners has initiated action to strengthen pre-service education in effective vaccine management by incorporating EVM, cold chain and immunisation supply content within its curricula for specific health cadres (Table 2).

**Table 2:** proposed training programmes at KMTC to incorporate EVM content

Training programme	Required skills and competencies
Medical engineering	Manage cold chain equipment
Nursing and midwifery	Manage immunisation programme
	Immunisation service providers
Environmental Health Sciences	Social mobilisation
	Cold chain management
Pharmaceutical Technologists	Vaccine safety
Medical Laboratory Technologistes	Diagnosing vaccine-preventable diseases
Clinical officers	Manage immunisation programme
Nutrition and dietetics	Child health
Health records and Information	Immunisation data management

The benefits of incorporation of EVM content in pre-service education are multiple: (1) Sustainability of capacity building initiatives through local institutions; (2) Increase in the pool of healthcare professionals across cadres with better understanding of EVM to support improvement actions within EPI; (3) Enhanced country ownership for immunisation supply chain; and (4) Improved practices in vaccine management.

## Conclusion

Increasing efforts have been made in the African Region during the past decade to strengthen the capacity of healthcare personnel responsible for delivering quality immunisation services to the public, by addressing both the pre-service and in-service training needs. While in-service training will remain necessary as new technologies and vaccines are being developed and guidelines formulated, pre-service health training institutions are crucial to deliver high quality training and education to future healthcare staff, as well as to support the in-service training in the country. The importance of health training institutions supporting the national immunisation programme has especially been proven important during the COVID-19 pandemic, when in-service training could be delivered by in-country staff only. With the introduction of new vaccines and technologies, increased complexity of immunisation programmes and current challenges with the COVID-19 pandemic, as well as the ambitious goals set by the Immunization Agenda 2030 [10], capacity building in immunisation remains at the forefront of well-functioning immunisation programmes. The different steps taken by KMTC to strengthen EPI teaching and learning can assist other health training institutions to integrate the content of the EPI prototype curriculum in their own institutional curricula adapted to the local context.

### What is known about this topic

- *Training needs assessment in some health training institutions in African countries have indicated gaps in EPI theory and practice;*
- *The EPI prototype curriculum can assist health training institutions with revising their institutional EPI curriculum;*
- *Few health training institutions have adapted their EPI curriculum*.

### What this study adds

- *KMTC used the EPI prototype curriculum to strengthen their institutional curriculum;*
- *The experience of KMTC outlines the different steps required to strengthen EPI pre-service training;*
- *Other health training institutions can learn from the experience of KMTC to strengthen EPI pre-service training*.

## References

[1] Swider S, Levin P, Ailey S, Breakwell S, Cowell J, McNaughton D (2006). Matching a graduate curriculum in public and community health nursing to practise competencies. Public Health Journal.

[2] Steinert Y, Mann K, Centeno A, Dolmans D, Spencer J, Gelula M (2006). A systematic review of faculty development initiatives designed to improve teaching effectiveness in medical education: BEME Guide No. 8. Med Teach.

[3] Mutabaruka E, Nshimirimana D, Goilav C, Meheus A (2005). EPI Training Needs Assessment in 12 African Countries, 2002-2004. WHO Regional Office for Africa, Communicable Diseases Bulletin for the African Region.

[4] Umar AS, Olatunji AO, Abiola AO, Yakubu A, Oche M (2011). Technical competence of tutors in pre-service health training institutions on Expanded programme on Immunisation in North Western Nigeria. Journal of Public Health and Epidemiology.

[5] Mutabaruka E, Dochez C, Nshimirimana D, Meheus A (2010). Evaluation of Mid-Level management training in immunisation in the African Region. East African Journal of Public Health.

[6] Masresha B, Dochez C, Bumgarner A, Pienkowski N, Mihigo R (2020). The World Health Organization African Regional training course for mid-level immunization managers: lessons and future directions. Pan African Medical Journal.

[7] Uskun E, Uskun SB, Uysalgenc M, Yagiz M (2008). Effectiveness of a training intervention on immunisation to increase knowledge of primary healthcare workers and vaccination coverage rates. Public Health.

[8] World Health Organization (WHO) Expanded programme on immunization prototype curriculum for nursing/midwifery schools in the WHO African Region.

[9] World Health Organization (WHO) Expanded programme on immunization prototype curriculum for medical schools in the WHO African Region.

[10] World Health Organization (WHO) Immunization Agenda 2030: A Global Strategy To Leave No One Behind.

